# Procyanidin B2 Alleviates Palmitic Acid-Induced Injury in HepG2 Cells via Endoplasmic Reticulum Stress Pathway

**DOI:** 10.1155/2021/8920757

**Published:** 2021-12-16

**Authors:** Yi-Ming Li, Shao-Yang Zhao, Huan-Huan Zhao, Bao-Hua Wang, Sai-Mei Li

**Affiliations:** ^1^The First School of Clinical Medicine, Guangzhou University of Chinese Medicine, Guangzhou 510000, China; ^2^The First Affiliated Hospital of Guangzhou University of Chinese Medicine, Guangzhou 510000, China; ^3^Nutrition Department, Linyi People's Hospital, Linyi 276000, China

## Abstract

Nonalcoholic fatty liver disease (NAFLD) is the hepatic manifestation of the metabolic syndrome featuring ectopic lipid accumulation in hepatocytes. NAFLD has been a severe threat to humans with a global prevalence of over 25% yet no approved drugs for the treatment to date. Previous studies showed that procyanidin B2 (PCB2), an active ingredient from herbal cinnamon, has an excellent hepatoprotective effect; however, the mechanism remains inconclusive. The present study aimed to investigate the protective effect and underlying mechanism of PCB2 on PA-induced cellular injury in human hepatoma HepG2 cells. Our results showed that PA-induced oxidative stress, calcium disequilibrium, and subsequent endoplasmic reticulum stress (ERS) mediated cellular injury, with elevated protein levels of GRP78, GRP94, CHOP, and hyperphosphorylation of PERK and IRE1*α* as well as the increased ratio of Bax/Bcl-2, which was restored by PCB2 in a concentration-dependent manner, proving the excellent antiapoptosis effect. In addition, 4-phenylbutyric acid (4-PBA), the ER stress inhibitor, increased cell viability and decreased protein levels of GRP78 and CHOP, which is similar to PCB2, and thapsigargin (TG), the ER stress agonist, exhibited conversely meanwhile partly counteracted the hepatic protection of PCB2. What is more, upregulated protein expression of p-IKK*α*/*β*, p-NF-*κ*B p65, NLRP3, cleaved caspase 1, and mature IL-1*β* occurred in HepG2 cells in response to PA stress while rescued with the PCB2 intervention. In conclusion, our study demonstrated that PA induces ERS in HepG2 cells and subsequently activates downstream NLRP3 inflammasome-mediated cellular injury, while PCB2 inhibits NLRP3/caspase 1/IL-1*β* pathway, inflammation, and apoptosis with the presence of ERS, thereby promoting cell survival, which may provide pharmacological evidence for clinical approaches on NAFLD.

## 1. Introduction

Nonalcoholic fatty liver disease (NAFLD) is a chronic pathological syndrome caused by excessive lipid accumulation in hepatocytes and steatosis [1, 2], including a spectrum from simple steatosis to nonalcoholic steatohepatitis (NASH), and may progress to cirrhosis and hepatocellular carcinoma [1, 3]. NAFLD is part of a multisystemic metabolic disorder associated with obesity, type 2 diabetes, and hyperlipidemia [4]. The disease has a global prevalence of over 25% and poses a serious threat to humans, but to date there are no approved therapeutic drugs [4–6].

Elevated circulating free fatty acids (FFAs) derived from adipose contribute to hepatic lipid accumulation [7–10]. Lipotoxicity of FFA and its metabolites leads to mitochondrial dysfunction, oxidative stress, endoplasmic reticulum stress, and apoptosis [1, 11, 12], thereby contributing to the development of NASH. Fatty acids are chemically classified as saturated and unsaturated [13], and palmitic acid (PA), the most common circulating saturated fatty acid [14], has been shown to be highly toxic to various cell lines. Previous studies have shown that human hepatoma HepG2 cells exposed to PA are susceptible to oxidative stress and apoptosis [15]; Ca^2+^ disequilibrium, ERS, and subsequent apoptosis occurred in OUMS-29 (human hepatocyte line) and Huh7 (human hepatocellular carcinoma-derived cell line) cells in response to PA stimulation [16]; PA-induced apoptosis in mouse hepatocyte AML-12 cells accompanied by ERS and activation of the NOD-like receptor family pyrin domain-containing 3 (NLRP3) inflammasome [17], and an in vitro inflammatory model of AML-12 cells were established in another study [18]. In addition, PA leads to inflammation in rat cardiomyocyte H9c2 and apoptosis in mouse pancreatic *β*-cell NIT-1 due to its cytotoxicity [19, 20].

ERS plays a pivotal role in the pathogenesis of NAFLD. Transient deleterious stimulation to the endoplasmic reticulum (ER) causes protein misfolding, ERS, and activation of the unfolded protein response (UPR) to resolve protein folding defects and reestablish the ER homeostasis initially, and conversion to apoptosis upon sustained or massive ERS [21]. ER chaperones, mainly glucose-regulated proteins 78 (GRP78) and 94 (GRP94), dissociate from three ER transmembrane transducers: the protein kinase RNA-like endoplasmic reticulum kinase (PERK), inositol-requiring enzyme 1*α* (IRE1*α*), and activating transcription factor 6*α* (ATF6*α*). PERK phosphorylates eukaryotic initiation factor 2*α* (eIF2*α*), which stops mRNA translation in response to stress. Phosphorylation of IRE1*α* activates transcription factors XBP1s to enhance both ER protein folding and protein degrading. The activation of ATF6*α* implicates in protein degradation as well. Three pathways disturb Ca^2+^ equilibrium in a Bcl-2 family-dependent manner and upregulate downstream targets [22], particularly C/EBP homologous protein (CHOP), triggering ERS-mediated apoptosis. Briefly, ERS is broadly involved in biological processes such as calcium and redox homeostasis, inflammatory response, activation of the NLRP3 inflammasome, and apoptosis [23].

There is conclusive evidence that palmitic acid (PA) induces ERS and further apoptosis in hepatocytes, and related pharmaceutical studies are well documented. The active ingredients of traditional Chinese decoctions and traditional herbal medicines have also had therapeutic implications in clinical trials and pharmacodynamics. Procyanidin B2 (PCB2), an active ingredient from herbal cinnamon (*Cinnamomum cassia*) [24], has demonstrated the beneficial effects of antioxidation, anti-inflammation, and antiapoptosis in mammals with NAFLD and other metabolic diseases [25–27]. Yet, research on whether and how PCB2 impacts ERS-mediated injury in PA-induced hepatocytes remains a gap. Hereby, we performed experiments to illustrate the subjects. Using PA-induced HepG2 cells as a model, the effects of PCB2 on ERS and NLRP3 inflammasome were investigated in vitro to provide pharmacological evidence for therapeutic strategies for NAFLD.

## 2. Materials and Methods

### 2.1. Reagents

**Figure 1:**
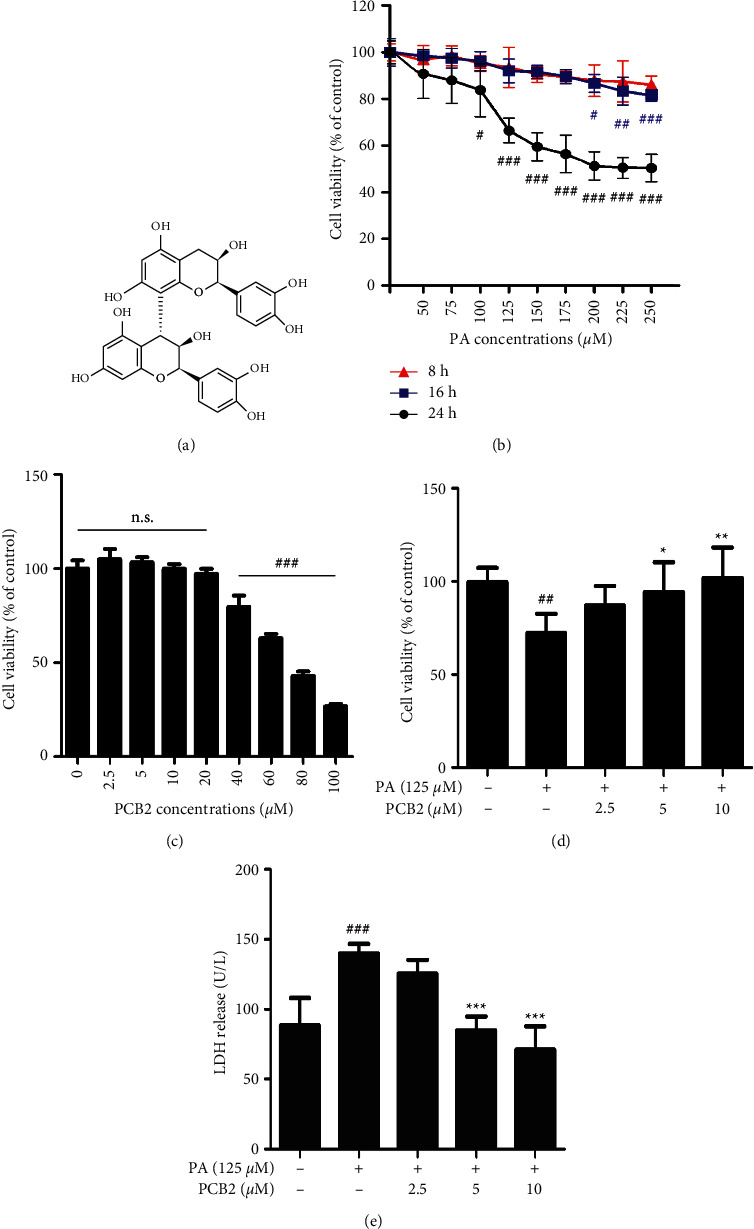
PCB2 protects HepG2 cells from PA-induced cell injury. (a) The chemical structure of PCB2. (b) HepG2 cells were treated with various concentrations of palmitic acid (from 50 to 250 *μ*M) for 8, 16, and 24 h respectively. Cell viability was measured using CCK-8 and normalized to control (%). (c) Cytotoxicity of PCB2 (from 2.5 to 100 *μ*M) for 24 h. (d) HepG2 cells were exposed to PA (125 *μ*M) and treated by PCB2 (2.5, 5, and 10 *μ*M) for 24 h. (c) HepG2 cells were treated as in (d), and (e) LDH released in the supernatant of cells was measured with a detection kit. The data are presented as means ± S.D. ^##^*P* < 0.01 and ^###^*P* < 0.001 vs control, ^*∗*^*P* < 0.05, ^*∗∗*^*P* < 0.01, and ^*∗∗∗*^*P* < 0.001 vs model group.

Procyanidin B2 (CAS: 29106-49-8, the chemical structure, see Figure 1(a)) and thapsigargin (TG) were obtained from Shanghai Yuanye Bio-Technology Co., Ltd (Shanghai, China). Cell Counting Kit-8 (CCK-8) was purchased from Dojindo Laboratories (Dojindo, Kumamoto, Japan). Detection kits of lactate dehydrogenase (LDH), superoxide dismutase (SOD), malondialdehyde (MDA), and calcium (Ca^2+^) were purchased from Nanjing Jiancheng Bioengineering Institute (Nanjing, Jiangsu Province, China). Annexin V-FITC/PI Kit was obtained from MultiSciences Biotech Co., Ltd (Hangzhou, Zhejiang Province, China). Terminal deoxynucleotidyl transferase (TdT)-mediated dUTP-biotin nick end-labeling (TUNEL) and ER-Tracker Red staining kits were purchased from Beyotime Biotechnology Co., Ltd (Shanghai, China). 4′,6-diamidino-2-phenylindole (DAPI), 2′,7′-dichlorofluorescein diacetate (DCFH-DA), and Hoechst 33342 were obtained from Sigma-Aldrich (Merck KGaA, Darmstadt, Germany). 4-Phenylbutyric acid (4-PBA) was obtained from Shanghai Yien Chemical Technology Co., Ltd (Shanghai, China). Antibodies for p-IRE1 (ab124945, 1 : 1000) and NLRP3 (ab263899, 1 : 1000) were purchased from Abcam (Cambridge, UK); p-PERK (bs-3330R, 1 : 1000) was purchased from Beijing Biosynthesis Biotechnology Co., Ltd. (Beijing, China); IL-1*β* (sc-12742, 1 : 200) was obtained from Santa Cruz Biotechnology, Inc. (Dallas, Texas, USA); and GRP78 (#3177, 1 : 1000), GRP94 (#2104, 1 : 1000), PERK (#5683, 1 : 1000), p-eIF2*α* (#3398, 1 : 1000), eIF2*α* (#5324, 1 : 1000), IRE1*α* (#3294, 1 : 1000), CHOP (#2895, 1 : 1000), cleaved caspase 1 (#4199, 1 : 1000), Bcl-2 (#3498, 1 : 1000), Bax (#2772, 1 : 1000), p-IKK*α*/*β* (#2697, 1 : 1000), IKK*β* (#8943, 1 : 1000), p-NF-*κ*B p65 (#3033, 1 : 1000), NF-*κ*B p65 (#8242, 1 : 1000), and GAPDH (#5174, 1 : 2000) were obtained from Cell Signaling Technology (Danvers, MA, USA).

### 2.2. Cell Culture

The human hepatoma HepG2 cell line was purchased from the Type Culture Collection Cell Bank, Chinese Academy of Science (Shanghai, China). Cells were maintained in Dulbecco's modified Eagle medium (DMEM; Gibco, CA, USA) supplemented with 10% (v/v) heat-inactivated fetal bovine serum (FBS; Biological Industries, Israel) and 1% (v/v) penicillin-streptomycin (PS, Gibco) in a humidified 5% CO_2_ incubator at 37°C.

### 2.3. Establishment of Injured HepG2 Model and Cytotoxicity of PCB2

HepG2 cells were exposed to various concentrations of palmitic acid (50, 75, 100, 125, 150, 175, 200, 225, and 250 *μ*M) for 8, 16, and 24 h, respectively. When the cell viability decreased to 60%–70%, the appropriate injured cell model was established. Then, based on the PA exposure duration, the cytotoxicity of PCB2 from 2.5 to 100 *μ*M was assessed.

### 2.4. Sample Treatment

HepG2 cells were divided into control, model, and PCB2 groups (low, medium, and high concentrations). The control group was cultured in DMEM (4.5 g/L D-glucose) without drug intervention. The model group was exposed to palmitic acid (125 *μ*M). The PCB2 groups were co-treated with various concentrations of PCB2 (2.5, 5, and 10 *μ*M) and palmitic acid. The drug exposure duration was 24 h for most experiments. Except where otherwise stated, all experiments were done at the same condition.

### 2.5. Cell Viability

Cell viability was determined using the Cell Counting Kit-8. Briefly, cells were seeded in 96-well plates (5000 cells/well) for 24 h. Then, the supernatant was replaced by culture medium containing various concentrations of PCB2 (with or without PA) and the plates were put back to the incubator for another 24 h. Next, the supernatant was replaced by CCK-8 solution (10 *μ*L CCK-8 in 90 *μ*L DMEM per well) and incubated for 2 h. The optical density (OD) values were measured at 450 nm using a microplate reader (Thermo Fisher Scientific, MA, USA). Cell viability (%) = (OD (treatment) – OD (blank))/(OD (control) – OD (blank)) × 100%.

### 2.6. LDH Release

HepG2 cells were exposed to PCB2 with or without PA as above. Then, a commercial kit was used for the detection of LDH release from the cells, according to the manufacturer's instructions. Absorbance was measured at 450 nm. LDH (U/L) = (OD (treatment) – OD (blank))/(OD (standard) – OD (blank)) × 0.2 (mmol/L) × 1000.

### 2.7. Annexin V/PI Staining

HepG2 cells were treated as shown previously after 12 h incubation in 6-well plates with an initial density of 2 × 10^5^ cells per well. All the operations simply followed the instructions. First, cells were harvested with the corresponding supernatants. Then, cells were washed twice with cold phosphate-buffered saline (PBS, Gibco) after centrifugation (500×g, 5 min). Next, cells were suspended in binding buffer, stained with Annexin V-FITC and PI for 5 min at room temperature, and detected within an hour with BD FACSCelesta (BD Biosciences, NJ, USA), and data were analyzed using FlowJo Software (BD, version: 10.7.2).

### 2.8. TUNEL Staining

According to the instructions of the One-step TUNEL Apoptosis Assay Kit, cells were grown on cover glasses (NEST, Wuxi, Jiangsu Province, China) placed at the bottom of a 24-well plate (3 × 10^4^ cells/well) and exposed to PA with or without PCB2 as described. The supernatant was removed, fixed by 4% paraformaldehyde for 30 min, permeabilized for 5 min with PBS containing 0.3% Triton X-100, and incubated with TUNEL working solution (mixed 5 *μ*L terminal deoxynucleotidyl transferase and 45 *μ*L fluorescent labeling solution for each sample) in the dark for 60 min at 37°C. The cover glasses were washed 3 times and then stained with DAPI solution (0.5 *μ*g/mL in PBS) for 1 min. The cover glasses were washed once more and placed in drying oven until desiccated. Images were captured using a fluorescence microscope (Olympus, Tokyo, Japan), and the relative intensity of fluorescence was measured using Image-Pro Plus Software (Media Cybernetics, Inc., MD, USA, version: 6.0).

### 2.9. Intracellular Reactive Oxygen Species (ROS) Detection

We used a fluorescent probe—DCFH-DA—to monitor intracellular ROS of HepG2 cells induced by PA. Cells were cultured as mentioned in the TUNEL staining assay except for the use of cover glasses, and the drug exposure duration was shortened to 12 hours. DCFH-DA was diluted in the culture medium as the final concentration was reduced to 10 *μ*M, with a 30 min incubation in the incubator. Then, gentle wash by PBS and re-incubation by DMEM are needed since it is essential to make the background clear. Observation of the highlighted green fluorescence was performed on the fluorescence microscope (Olympus).

### 2.10. Intracellular SOD/MDA/Ca^2+^ Detection

HepG2 cells were grown in 100 mm dishes and treated for 24 h. Cells were suspended in cold PBS and ruptured by ultrasonic instrument (Shunma Tech, Jiangsu Province, China) at a low power. Thus we acquired limpid supernatant and then determined protein concentrations with a Bicinchoninic Acid (BCA) Assay Kit (TransGen Biotech, Beijing, China) and used detection kit of SOD for evaluation. MDA and Ca^2+^ levels were measured in the same way. Operations were followed by the instructions of the kits. For SOD detection, after the determination of protein contents we need to dilute the supernatant to a proper concentration, making SOD inhibitory rate vary around 40%–60%.

The SOD inhibitory rate (%) = {(OD (control) – OD (control blank) – (OD (treatment) – OD (treatment blank))}/(OD (control) – OD (control blank)) × 100%.

The SOD activity (U/mg) = SOD inhibitory rate/50% × (reactive system (*μ*l)/(dilute proportion))/protein contents (mg/mL).

The MDA contents (nmol/mg) = (OD (treatment) – OD (blank))/(OD (standard) – OD (blank)) × MDA standard (10 nmol/ml)/protein contents (mg/mL).

The calcium contents (*μ*mol/mg) = (OD (treatment) – OD (blank))/(OD (standard) – OD (blank)) × calcium standard (10 nmol/ml)/protein contents (mg/mL).

### 2.11. ER-Tracker Red and Hoechst 33342 Staining

HepG2 cells were treated as in the ROS detection assay. 5 *μ*L ER-Tracker Red was mixed with 10,000 *μ*L ER-Tracker Red diluent preheated in the 37°C water bath. Then, a total of 10,005 *μ*L working solution took the place of culture medium and incubated at 37°C in the dark for 30 min. Next, each well was washed by PBS, followed by Hoechst 33342 (1 *μ*g/ml in PBS) staining for 1 min. The cells were observed using a fluorescence microscope under excitation wavelength of 587 nm/emission wavelength of 615 nm for ER-Tracker Red staining and excitation wavelength of 346 nm/emission wavelength of 460 nm for Hoechst 33342 staining.

### 2.12. Quantitative Real-Time Polymerase Chain Reaction (PCR) Analysis

HepG2 cells were cultured in 60 mm dishes with 1 × 10^6^ cells per vessel and exposed to drugs for 8 h. RNA was extracted from cells using RNA Easy Fast Tissue/Cell Kit (Tiangen Biotech, Beijing, China). The RNA concentration from each sample was measured using NanoDrop 2000 (Thermo Fisher Scientific). Samples with a concentration of 200–500 ng/*μ*L and an OD_260/280_ value of 2.0–2.5 were used in subsequent experiments. A total of 1.5 *μ*g RNA from each sample was used for reverse transcription and cDNA synthesis with FastKing gDNA Dispelling RT SuperMix (Tiangen Biotech). The real-time PCR (20 *μ*L), containing 10 *μ*L 2 × Talent qPCR PreMix, 0.3 *μ*L primers (forward and reverse, respectively, 20 *μ*mol/L), 1.0 *μ*L cDNA template, 0.4 *μ*L ROX Dye (50 ×), and 8.0 *μ*L RNase-free ddH_2_O, was performed on ABI 7500 System (Applied Biosystems, MA, USA). The PCR amplification procedures were set as manual states: predenaturation at 95°C for 3 min followed by 40 cycles at 95°C for 5 s and 60°C for 32 s. The mRNA levels were normalized to *β*-actin and assessed using the 2^−∆∆Ct^ method. The primer sequences for genes of interest are listed in Table 1.

### 2.13. Western Blotting

At the end of the drug intervention, HepG2 cells were vortexed in cold RIPA buffer with protease and phosphatase inhibitor for 10 min. Total proteins were extracted after centrifugation (14000 rpm, 10 min, 4°C), and protein concentrations were determined using a BCA Assay Kit (TransGen Biotech). Protein samples from each group were subjected to an SDS-PAGE system and transferred to polyvinylidene fluoride (PVDF) membranes (Millipore, Billerica, MA, USA). The membranes were blocked by 5% (w/v) skim milk at room temperature for 60 min, then washed 3 times with Tris-buffered saline containing 0.1% Tween-20 (TBST), and incubated with primary antibody at 4°C overnight with slight shaking. The membranes were washed 3 times and incubated with secondary antibody at room temperature for 2 h. Finally, the membranes were washed another 3 times and exposed to Immobilon Western Chemiluminescent HRP Substrate (Millipore). Protein bands were gauged with ChemiDoc Imaging Systems (Bio-Rad, CA, USA).

### 2.14. Statistical Analysis

All experiments were performed at least three times with triplicate using a one-way analysis of variance. Statistical analyses were performed using GraphPad Prism Software (GraphPad Software; San Diego, CA, USA, version: 8.0). The data were expressed as means ± standard deviation (S.D.). Comparisons between different groups were carried out with Student's *t*-test as appropriate. A value of *P* < 0.05 was considered to be significant.

## 3. Results

### 3.1. PCB2 Protects HepG2 Cells from PA-Induced Cell Injury

Previous studies have shown that PA causes damage to HepG2 cells, so we tried to establish the PA-induced injured HepG2 model in a concentration- and time-dependent manner. Cell viability of the groups exposed to PA decreased gradually with concentration increased. For 8h-treated groups, there was no significance v.s. control. The cell viability of 16h-treated groups was all above 80%, and that of 24h-treated groups decreased further to 60%–70% importantly, cell viability of 125 *μ*M PA was 66.48 ± 4.9% (*P* < 0.001 vs control, Figure 1(b)). The data in Figure 1(c) showed that intervention of PCB2 from 2.5 to 100 *μ*M led to decrease in cell viability in a concentration-dependent manner, and when it is below 20 *μ*M of PCB2, there was no significance vs control. Next, we used CCK-8, a modified method of MTT assay, to assess the protective effect of PCB2 against PA. The data showed that PA (125 *μ*M) caused significant cell damage (cell viability decreased to 72.55 ± 9.3%, *P* < 0.01 vs control), whereas PCB2 (2.5, 5, and 10 *μ*M) reversed the damage in a concentration-dependent manner, with cell viability rising to 102.28 ± 15.0% in the high concentration group (*P* < 0.01 vs model, Figure 1(d)). Furthermore, we detected LDH release from injured cells to assess cytotoxicity following drug exposure. As shown in Figure 1(e), LDH released from model cells was dramatically inflated to 139.93 ± 6.15 U/L (*P* < 0.001 vs control), whereas PCB2 (5 and 10 *μ*M) significantly scaled down the release and remedied the cytotoxicity (84.82 ± 9.0 and 71.13 ± 15.16 U/L, respectively, *P* < 0.001 vs model). Briefly, our results indicate that PCB2 is well protected against PA-induced damage to HepG2 cells.

### 3.2. PCB2 Inhibits Apoptosis in PA-Stressed HepG2 Cells

**Figure 2:**
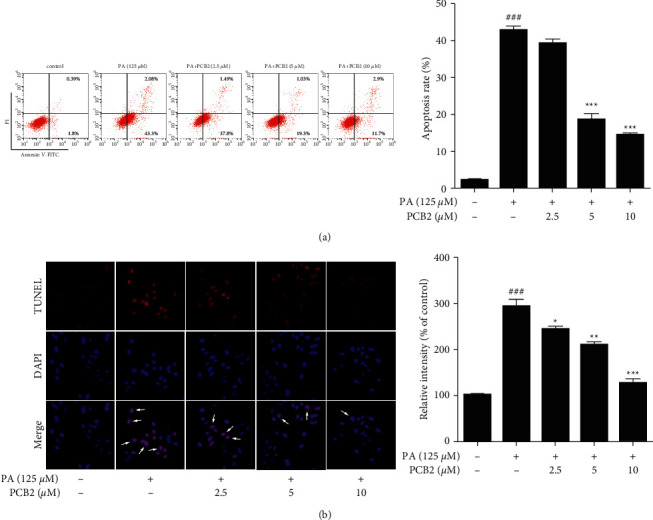
PCB2 inhibits apoptosis in PA-stressed HepG2 cells. (a) HepG2 cells were exposed to PA (125 *μ*M) and treated by PCB2 (2.5, 5, and 10 *μ*M) for 12 h. Apoptosis rate was determined with Annexin V-FITC/PI staining on a flow cytometer. (b) HepG2 cells were exposed to PA with or without PCB2 for 24 h. Fixed cells were incubated with TUNEL (red) and DAPI (blue) solution, respectively, and relative fluorescence intensity was measured. Scale bar = 50 *μ*m. Typical apoptotic cells were marked with white arrows. The data are presented as means ± S.D. ^###^*P* < 0.001 vs control, ^*∗*^*P* < 0.05, ^*∗∗*^*P* < 0.01, and ^*∗∗∗*^*P* < 0.001 vs model group.

To determine the apoptosis in HepG2 cells accurately, quantitative assays were performed on a flow cytometer using Annexin V/PI staining. Translocation of phosphatidylserine occurred in early apoptotic cells whose cytomembranes were stained with Annexin V only. Late apoptotic cells had incomplete cell membranes, resulting in nuclei stained with PI. Cells stained by both Annexin V and PI were considered necrotic or fragmented. Dot plots (Figure 2(a)) showed percentage of apoptotic cells (early and late apoptotic cells) in each group—it was 2.19% in control, 45.38% in model (*P* < 0.001 vs control), 39.29% in low concentration (n.s.), 20.33% in medium concentration (*P* < 0.001 vs model), and 14.6% in high concentration (*P* < 0.001 vs model), respectively. Moreover, to observe apoptosis directly, TUNEL staining was performed on HepG2 cells. This was based on the ability of TdT to catalyze the addition of dUTPs labeled with Cy3 to free 3′-hydroxyl termini of DNA, thereby enabling visualization of nuclei containing fragmented DNA cleaved in the biological process of apoptosis. Images of red (TUNEL) and blue (DAPI) fluorescence were captured (Figure 2(b)), and the relative intensity of the fluorescence was measured. The model group had more red apoptotic cells with higher relative fluorescence intensity than the control group (*P* < 0.001), which was significantly prevented by PCB2 treatment. These results are in line with flow cytometry analysis, suggesting that PCB2 can effectively mitigate apoptosis in PA-stressed HepG2 cells.

### 3.3. PCB2 Mitigates Oxidative Stress and Calcium Disequilibrium in PA-Induced HepG2 Cells

**Figure 3:**
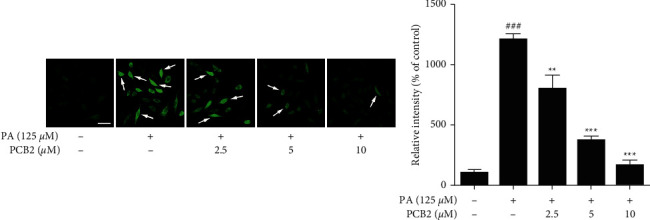
PCB2 inhibits ROS formation in PA-induced HepG2 cells. Cells were exposed to PA (125 *μ*M) and treated by PCB2 (2.5, 5, and 10 *μ*M) for 12 h. Intracellular ROS detected with DCFH-DA (green) and relative fluorescence intensity were measured. Scale bar = 50 *μ*m. Typical cells with excessive ROS were marked by white arrows. The data are presented as means ± S.D. ^###^*P* < 0.001 vs control, ^*∗∗*^*P* < 0.01 and ^*∗∗∗*^*P* < 0.001 vs model group.

To assess the antioxidation effect of PCB2, indicators associated with oxidative stress, including ROS, MDA, and SOD, were measured. First, a cell-permeable fluorescent probe—DCFH-DA, blazes upon oxidation and can therefore be of great use in the sensitive and rapid quantitation of intracellular ROS. As shown in Figure 3, the more the ROS formation, the more prominent the captured green fluorescence, so PA (125 *μ*M)-induced HepG2 cells were seen to be dramatically immersed in green and the relative fluorescence intensity elevated 10 times more than control (*P* < 0.001). After 12 h treatment of PCB2 (2.5, 5, and 10 *μ*M), the ROS were effectively reduced in a concentration-dependent manner (*P* < 0.01 and *P* < 0.001 vs model). Likewise, MDA was assayed to examine lipid peroxidation and cell damage subjected to ROS attack, and as expected, much more MDA was formed in PA-stressed cells than in control cells (*P* < 0.01, Table 2) and showed a gradual decrease in PCB2-treated cells (evident in 10 *μ*M of PCB2, *P* < 0.01), consistent with the intracellular ROS assay. We then measured SOD, one of the major antioxidant enzymes, whose activity controls the formation of ROS and therefore limits its potential toxicity and regulates broad aspects of cellular life. The data in Table 2 demonstrated that PCB2 restored the decrease in SOD activity induced by PA markedly (*P* < 0.01 and *P* < 0.001 vs model).

Calcium and redox homeostasis play key roles in cell life and death; therefore, we applied the tool of detection of Ca^2+^ in the cytoplasm. Also in Table 2, we observed a significant increase in cells exposed to PA (*P* < 0.001 vs control) and a decrease in cells treated with PCB2 (*P* < 0.01 and *P* < 0.001 vs model). Taken together, the evidence from these assays reveals that PCB2 has the ability to prevent oxidative stress and Ca^2+^ disequilibrium in PA-induced HepG2 cells.

### 3.4. PCB2 Rescues PA-Induced Endoplasmic Reticulum Dysfunction in HepG2 Cells

**Figure 4:**
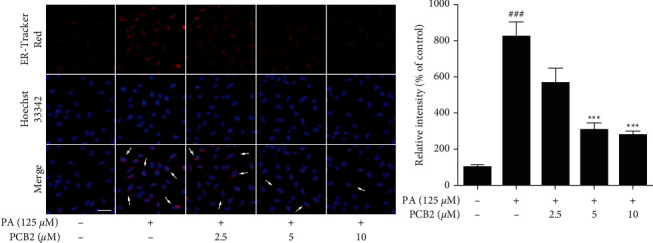
PCB2 rescues PA-induced endoplasmic reticulum dysfunction in HepG2 cells. Cells were exposed to PA (125 *μ*M) and treated by PCB2 (2.5, 5, and 10 *μ*M) for 12 h and then incubated with ER-Tracker Red (red) and Hoechst 33342 (blue) solution, respectively, and relative fluorescence intensity was measured. Scale bar = 50 *μ*m. Typical cells were marked with white arrows. The data are presented as means ± S.D. ^###^*P* < 0.001 vs control, ^*∗∗∗*^*P* < 0.001 vs model group.

As the above results showed Ca^2+^ perturbations in the cells after treatment, we were increasingly interested in observing the damage to the ER caused by PA (125 *μ*M). We therefore assessed this with ER-Tracker Red, based on the ability of glibenclamide (glyburide) to bind specific receptors on ER, followed by Hoechst 33342 staining, which is cell-permeable, nuclei-specific, and useful for studying apoptosis in live cells. The former glowed red fluorescence labeling ER, while the latter emitted blue labeling nuclei of apoptotic cells (Figure 4). The relevant mean fluorescence intensity was calculated for each group, and the results were consistent with the detection of Ca^2+^ level (*P* < 0.001).

### 3.5. PCB2 Performs Antiapoptosis Effect through Bcl-2 Family in PA-Induced HepG2 Cells

**Figure 5:**
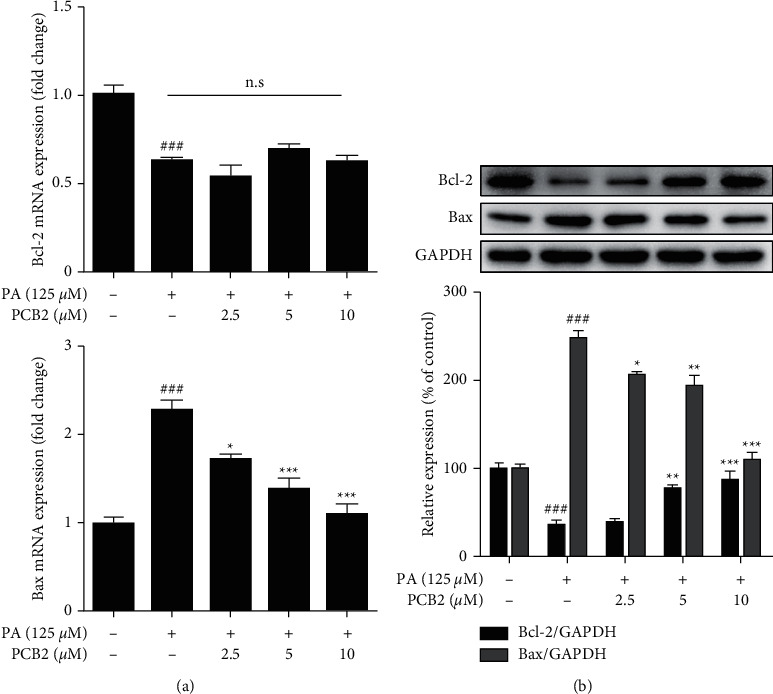
PCB2 performs anti-apoptosis effect through Bcl-2 family in PA-induced HepG2 cells. (a) HepG2 cells were exposed to PA (125 μM) and treated by PCB2 (2.5, 5 and 10 μM) for 8 h. The mRNA levels of Bcl-2 and Bax were examined by qPCR. (b) HepG2 cells were exposed to drugs for 24 h. The protein levels of Bcl-2 and Bax were examined by western blotting. Data are presented as mean ± S.D. ^###^*P* < 0.001 vs control, ^∗^*P* < 0.05, ^∗∗^*P* < 0.01, and ^∗∗∗^*P* < 0.001 vs model group.

One of the vital regulators to apoptosis, known as the “Bcl-2 family,” whose members (Bcl-2, Bcl-XL, Mcl-1, etc.) prevent apoptosis, should be balanced with pro-apoptotic ones such as Bax, Bak, and Bad. Under certain stimuli, such as PA exposure as reported in numerous studies, it would lead to a boost in the Bax/Bcl-2 ratio and thus promote apoptosis. Herein, we determined the expressions of Bcl-2 and Bax at both transcriptional and translational levels. In PA (125 *μ*M)-induced HepG2 cells, mRNA and protein expression of Bax (Figures 5(a) and 5(b)) was significantly increased compared with control (*P* < 0.001) and significantly decreased in PCB2 (2.5, 5, and 10 *μ*M)-treated cells (*P* < 0.05 and *P* < 0.01 and *P* < 0.001 vs model). As for Bcl-2, the protein expression was significantly reduced in PA-exposed cells compared with control cells (*P* < 0.001) while exhibited a gradient elevation after PCB2 treatment (evident in 5 and *10 μ*M of PCB2, *P* < 0.01 and *P* < 0.001 vs model). However, PCB2 treatment could not affect mRNA expression. All the above results suggest the antiapoptosis effect of PCB2 in HepG2 cells against PA is associated with Bcl-2 family.

### 3.6. PCB2 Alleviates Endoplasmic Reticulum Stress in PA-Induced HepG2 Cells

**Figure 6:**
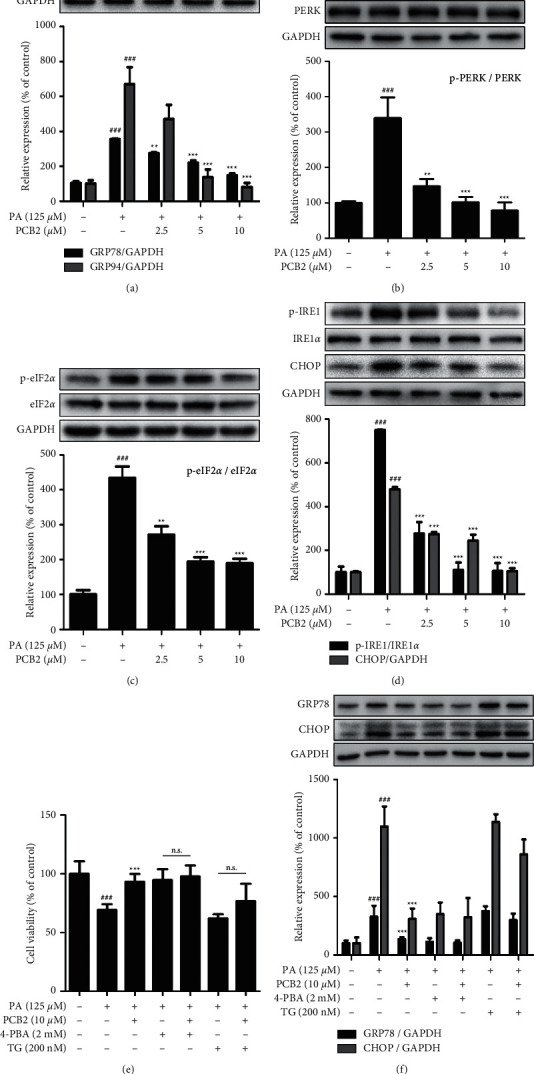
PCB2 alleviates endoplasmic reticulum stress in PA-induced HepG2 cells. Cells were exposed to PA (125 *μ*M) and treated by PCB2 (2.5, 5, and 10 *μ*M) for 24 h. The protein levels were examined by Western blotting. (a) The protein levels of GRP78 and GRP94. (b) The protein levels of p-PERK and PERK. (c) The protein levels of p-eIF2*α* and eIF2*α*. (d) The protein levels of p-IRE1, IRE1*α,* and CHOP. (e) Cell viability of model, PCB2 (10 *μ*M), and ERS pathway inhibitor/agonist-related groups. (f) HepG2 cells were treated as in (e), and the relative protein levels of GRP78 and CHOP were measured by Western blot. ^###^*P* < 0.001 vs control, ^*∗∗*^*P* < 0.01 and ^*∗∗∗*^*P* < 0.001 vs model group.

Furthermore, we applied Western blotting to assess PA (125 *μ*M)-induced expression of target proteins associated with ERS in HepG2 cells. Upon PA stress, glucose-regulated proteins 78 (GRP78) and 94 (GRP94) dissociate and bind to misfolded or unfolded proteins, thus promoting phosphorylation of PERK, IRE1*α*, and the downstream eIF2*α*, leading to activation of CHOP, a key trigger for ERS-mediated apoptosis. The data in Figure 6(a) show that levels of both GRP78 and GRP94 were elevated in model cells (*P* < 0.001 vs control) and were restored by PCB2 (*P* < 0.01 and *P* < 0.001 vs model). In Figures 6(b)–6(d), the phosphorylation levels of PERK, eIF2*α*, and IRE1*α* were upregulated by PA (*P* < 0.01 and *P* < 0.001 vs control) and downregulated by PCB2 (*P* < 0.05 and *P* < 0.01 and *P* < 0.001 vs model). In addition, CHOP expression was consistent with the above targets.

To determine the involvement of the protection of PCB2 to injured HepG2 cells induced by PA, 4-PBA, a compound that inhibits ER stress, was used to verify the protective effect. As shown in Figures 6(e) and 6(f), 4-PBA (2 mM) increased cell viability and decreased relative expression of crucial targets of ERS pathway—GRP78 and CHOP (*P* < 0.001 vs model group). PCB2 treatment (10 *μ*M) was similar to the co-treatment of PA and 4-PBA (n.s.). Furthermore, TG, an ER stress agonist, led to significant cell death and overexpression of GRP78 and CHOP (*P* < 0.001 vs control), as well as partly counteracted the hepatic protection of PCB2 when co-treated with it (n.s.). Overall, our data indicated that PCB2 mitigates endoplasmic reticulum stress in HepG2 cells against PA exposure, and the protective effect is ERS pathway-dependent.

### 3.7. PCB2 Suppresses ERS-Mediated NLRP3 Inflammasome Activation in HepG2 Cells

**Figure 7:**
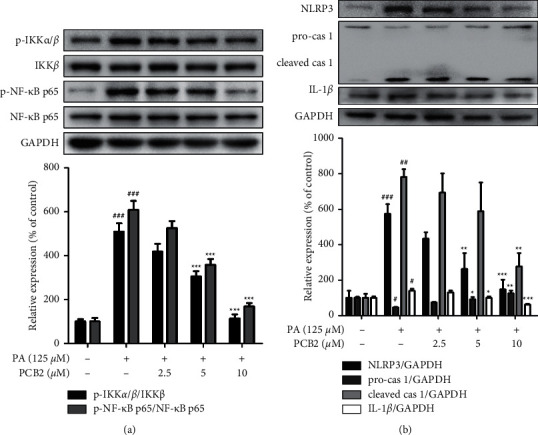
PCB2 suppresses ERS-mediated activation of NF-*κ*B pathway and NLRP3 inflammasome in HepG2 cells. Cells were exposed to PA (125 *μ*M) and treated by PCB2 (2.5, 5, and 10 *μ*M) for 24 h. The protein levels were examined by Western blotting. (a) The protein levels of p-IKK*α*/*β*, IKK*β*, p-NF-*κ*B p65, and NF-*κ*B p65. (b) The protein levels of NLRP3, pro-caspase 1, cleaved caspase 1, and cleaved IL-1*β*. ^#^*P* < 0.05, ^##^*P* < 0.01, and ^###^*P* < 0.001 vs control, ^*∗*^*P* < 0.05, ^*∗∗*^*P* < 0.01, and ^*∗∗∗*^*P* < 0.001 vs model group.

There is growing evidence that ERS-induced activation of the NLRP3 inflammasome underlies the pathology of various inflammatory diseases. To investigate whether PCB2 affects these pathological changes in HepG2 cells at the presence of ERS, we measured the relative expression of NLRP3, caspase 1, IL-1*β,* and components of the nuclear factor kappa B (NF-*κ*B) pathway. The results in Figure 7 showed that PA (125 *μ*M) resulted in substantial upregulation of p-IKK*α*/*β*, p-NF-*κ*B p65, NLRP3, cleaved caspase 1, and cleaved IL-1*β* levels compared with control (*P* < 0.001), while PCB2 contributed to concentration-dependent inhibition (*P* < 0.01 and *P* < 0.001 vs model). Collectively, PCB2 could restrain ERS-mediated NLRP3 inflammasome activation and inflammation in PA-induced HepG2 cells.

## 4. Discussion

Nonalcoholic fatty liver disease (NAFLD) is the most prevalent chronic liver disease worldwide and is considered the hepatic manifestation of the metabolic syndrome associated with obesity and type 2 diabetes [4], increasing the risk of cardiovascular disease and some types of cancer. NAFLD is characterized by the accumulation of ectopic lipids in hepatocytes [1], during which the elevated circulating FFA derived from adipose tissue is required. Increased influx of FFA to the liver leads to lipotoxicity promoting progressive NAFLD [9, 12].

To a certain extent, traditional Chinese medicine (TCM) has a wealth of experience and effectiveness in the treatment of NAFLD. In China, TCM is used in decoctions to treat patients with various symptoms [28], but uncertainty about their mechanisms and inconsistent efficacy in large-scale randomized controlled trials limit the application of decoctions. Notably, there has been progress in a large number of basic studies on extracts or active ingredients of single herbs, with silymarin and berberine already in phase 4 clinical studies approved by the US Food and Drug Administration [29]. Similar researches are in full swing for providing effective drugs on NAFLD. As an active ingredient extracted from traditional Chinese herbal Cinnamon (*Cinnamomum cassia*) [24], PCB2 offers excellent hepatic protection, but few pharmacodynamic studies are reported to date.

PA has been shown to be the most common circulating saturated fatty acid that causes hepatocyte apoptosis and is associated with ERS. Recent studies have revealed that ERS is a contributor to the development of NAFLD [1]. In response to PA stimulation, excessive unfolded or misfolded proteins overloaded in ER induce excessive ROS formation and Ca^2+^ disequilibrium, thus activating ERS pathways. Ca^2+^ massive influx to mitochondrion leads to more ROS production in turn [30, 31]. In this study, we found that PCB2 effectively inhibited cytosolic Ca^2+^ overload and ROS formation in PA-treated HepG2 cells, which is consistent with the previous study demonstrating that PCB2 inhibits FFA-induced oxidative stress via restoring mitochondrial membrane potential and scavenging ROS [25].

In the physiological state, the transmembrane proteins of ER, PERK, and IRE1*α* remain inactive and bind to the ER chaperone GRP78. As the bulk of unfolded proteins accumulate in ER, GRP78 dissociates from PERK- and IRE1*α*-activating downstream pathways, respectively, to reestablish cellular homeostasis. However, sustained or severe stimuli activate pro-apoptosis or inflammatory genes, resulting in irreversible cellular injury. At the same time, persistent UPR causes elevated protein expression of the ER chaperones GRP78 and GRP94 [32]. In this work, we observed that PCB2 downregulated the phosphorylation of PERK and IRE1*α* as well as the protein levels of GRP78, GRP94, and CHOP, revealing that PCB2 attenuates extensive PA-induced ERS through molecular chaperones, stress sensors, and transcription factor levels, consequently rescuing cellular injury. Yogalakshmi et al. showed that PCB2 restrained ERS by downregulating mRNA levels of PERK, IRE1*α*, and ATF-6 and protein levels of eIF2*α* and XBP1 in high-calorie diet-fed rats [33]. Furthermore, we used 4-PBA and TG, the ER stress inhibitor and agonist, respectively [34, 35], to assess the protective role of PCB2 in the context of PA-induced injury in HepG2 cells, indicating that ERS-dependent prevention could be involved in its protective effects. Previous and current experiments have confirmed the anti-ERS effects of PCB2 at multiple levels.

ER stress is implicated in complicated signaling pathways. The literature reports that TXNIP, a natural antagonist of thioredoxin (TRX), is an important molecule linking ERS and cell apoptosis—upon ERS, phosphorylated PERK and IRE1*α* activate thioredoxin-interacting protein (TXNIP) and NLRP3 inflammasome, which induce inflammation and cell death [36, 37]. The NLRP3 inflammasome is a multiprotein complex consisting of NLRP3, apoptosis-associated speck-like protein containing a CARD (ASC), and pro-caspase 1. Once the NLRP3 inflammasome activates, pro-caspase-1 cleaves pro-IL-1*β* into a mature form, and thus, activated IL-1*β* triggers inflammation and cell injury via NF-*κ*B pathway [38, 39]. Our experiments revealed that PCB2 inhibits the activation of the NLRP3 inflammasome and the increase in the Bax/Bcl-2 ratio, which is a key regulator of the mitochondrial apoptosis pathway, in a concentration-dependent manner. Flow cytometry, TUNEL, and Hoechst 33342 staining proved the protective effect of PCB2 against apoptosis induced by PA in vitro. Previous studies have shown that ERS is a crucial mechanism in cell death driven by PA [16, 17]; meanwhile, we did a preliminary but exploratory investigation of the effect of NLRP3 inflammasome activation regulated by PCB2 on ERS-mediated cell injury. Li Ma et al. reported that PCB2 significantly inhibited cold stimulation-induced NLRP3 inflammasome activation and IL-1*β* secretion in rats' liver [40]. Similarly, here we examined the protein expression of p-IKK*α*/*β*, p-NF-*κ*B p65, NLRP3, cleaved caspase 1, and mature IL-1*β*, proving the inhibition of PCB2. Yet, it remains poorly understood that if PCB2 functions in vivo as above, we will perform experiments using rodent models for further investigations.

## 5. Conclusions

**Figure 8:**
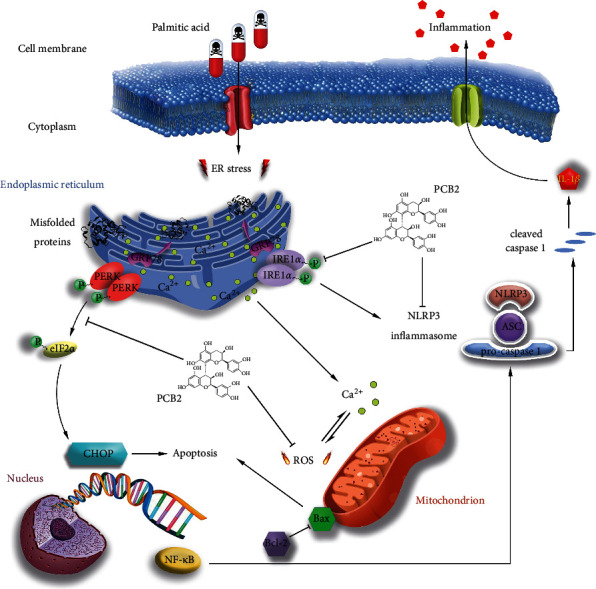
Mechanism of PCB2 against PA-induced injury in HepG2 cells.

This work reveals the mechanism by which PCB2 attenuates PA-induced injury in HepG2 cells. PA results in NLRP3 inflammasome-dependent cellular inflammatory response and mitochondrial-dependent apoptosis at the presence of ROS-mediated ERS. PCB2 intervention alleviates oxidative stress and endoplasmic reticulum stress while restoring antioxidant enzyme activities and Ca^2+^ equilibrium. Furthermore, PCB2 inhibits NF-*κ*B and NLRP3/caspase 1/IL-1*β* pathways associated with inflammation and Bax/Bcl-2-dependent cell apoptosis, thus promoting cell survival (Figure 8). All of these may provide preliminary pharmacological evidence for clinical treatment of NAFLD.

## Figures and Tables

**Table 1:** Primer sequences used in quantitative real-time PCR.

Gene	Forward (5′–3′)	Reverse (5′–3′)
*Bax*	GTTTTCTGACGGCAACTTCAACT	CCCATGATGGTTCTGATCAGTTC
*Bcl-2*	GTGTTCCGCGTGATTGAAGAC	TCCCAGAGGAAAAGCAACG
*β-Actin*	TCAAGATCATTGCTCCTCCTGAG	ACATCTGCTGGAAGGTGGACA

**Table 2:** Effect of PCB2 on SOD, MDA, and Ca^2+^ levels in PA-induced HepG2 cells

Groups	SOD (U/mg)	MDA (nmol/mg)	Ca^2+^ (*μ*mol/mg)
Control	**2980.011** **±** **140.288**	**1.165** **±** **0.106**	**0.181** **±** **0.01**
PA (125 *μ*M)	**2405.464** **±** **90.575**^**##**^	**1.521** **±** **0.032**^**##**^	**0.224** **±** **0.004**^**###**^
PA **+** PCB2 (2.5 *μ*M)	**2953.916** **±** **105.401**^*∗∗*^	**1.347** **±** **0.024**	**0.20** **±** **0.007**^*∗∗*^
PA **+** PCB2 (5 *μ*M)	**3045.872** **±** **114.002**^*∗∗*^	**1.321** **±** **0.022**	**0.196** **±** **0.009**^*∗∗∗*^
PA **+** PCB2 (10 *μ*M)	**3323.062** **±** **158.858**^*∗∗∗*^	**1.193** **±** **0.038**^*∗∗*^	**0.19** **±** **0.008**^*∗∗∗*^

HepG2 cells were exposed to PA (125 *μ*M) and treated by PCB2 (2.5, 5, and 10 *μ*M) for 24 h. SOD, MDA, and Ca^2+^ levels were detected by kits, respectively. The bold values are presented as means ± S.D.^##^*P* < 0.01 and^###^*P* < 0.001 vs control, ^*∗*^*P* < 0.05, ^*∗∗*^*P* < 0.01, and ^*∗∗∗*^*P* < 0.001 vs model groups For SOD level, control vs. PA: *P* = 0.0066; PA vs. 2.5*μ*M: *P* = 0.0089; PA vs. 5*μ*M: *P* = 0.0031; PA vs. 10*μ*M: *P* = 0.0002. For MDA level, control vs. PA: *P* = 0.0027; PA vs. 10*μ*M: *P* = 0.0053. For Ca^2+^ level, control vs. PA: P<0.0001; PA vs. 2.5*μ*M: *P* = 0.0014; PA vs. 5*μ*M: *P* = 0.0003; PA vs. 10*μ*M: P<0.0001. .

## Data Availability

All data are available from the corresponding author upon request.

## Abbreviations

NAFLD: Nonalcoholic fatty liver disease
NASH: Nonalcoholic steatohepatitis
FFA: Free fatty acids
PA: Palmitic acid
PCB2: Procyanidin B2
ER: Endoplasmic reticulum
ERS: Endoplasmic reticulum stress
UPR: Unfolded protein response
NLRP3: NOD-like receptor family pyrin domain-containing 3
GRP78: Glucose-regulated protein 78
GRP94: Glucose-regulated protein 94
PERK: Protein kinase RNA-like endoplasmic reticulum kinase
IRE1*α*: Inositol-requiring enzyme 1*α*
ATF6*α*: Activating transcription factor 6*α*
eIF2*α*: Eukaryotic initiation factor 2*α*
Bcl-2: B-cell lymphoma-2
Bax: Bcl-2-associated X protein
CHOP: C/EBP homologous protein
LDH: Lactate dehydrogenase
ROS: Reactive oxygen species
SOD: Superoxide dismutase
MDA: Malondialdehyde
TUNEL: Terminal deoxynucleotidyl transferase-mediated dUTP-biotin nick end-labeling
DAPI: 4′,6-diamidino-2-phenylindole
DCFH-DA: 2′,7′-Dichlorofluorescein diacetate
PCR: Polymerase chain reaction.
